# Generation of Vortex Optical Beams Based on Chiral Fiber-Optic Periodic Structures

**DOI:** 10.3390/s20185345

**Published:** 2020-09-18

**Authors:** Azat Gizatulin, Ivan Meshkov, Irina Vinogradova, Valery Bagmanov, Elizaveta Grakhova, Albert Sultanov

**Affiliations:** Department of Telecommunications, Ufa State Aviation Technical University, 450008 Ufa, Russia; mik.ivan@bk.ru (I.M.); vil-4@mail.ru (I.V.); bagmanov.valeriy@yandex.ru (V.B.); eorlingsbest@mail.ru (E.G.); tks@ugatu.ac.ru (A.S.)

**Keywords:** fiber Bragg gratings, chiral structures, orbital angular momentum, apodization, chirp, coupled modes theory

## Abstract

In this paper, we consider the process of fiber vortex modes generation using chiral periodic structures that include both chiral optical fibers and chiral (vortex) fiber Bragg gratings (ChFBGs). A generalized theoretical model of the ChFBG is developed including an arbitrary function of apodization and chirping, which provides a way to calculate gratings that generate vortex modes with a given state for the required frequency band and reflection coefficient. In addition, a matrix method for describing the ChFBG is proposed, based on the mathematical apparatus of the coupled modes theory and scattering matrices. Simulation modeling of the fiber structures considered is carried out. Chiral optical fibers maintaining optical vortex propagation are also described. It is also proposed to use chiral fiber-optic periodic structures as sensors of physical fields (temperature, strain, etc.), which can be applied to address multi-sensor monitoring systems due to a unique address parameter—the orbital angular momentum of optical radiation.

## 1. Introduction

Nowadays, the demand for broadband multimedia services is still growing, which leads to an increase of transmitted data volume as part of the development of the digital economy and the expansion of the range of services (video conferencing, telemedicine, online broadcasting, streaming, etc.). This circumstance requires the development of broadband access technologies in both wired (fiber optic, copper) and wireless networks, however, the existing ways to increase the throughput of communication systems (based on the use of time, frequency, polarization, space as a multiplexing domains) face the theoretical Shannon’s throughput limit, which forces researchers to look for alternative physical parameters of electromagnetic (EM) waves that can be used to transmit information. For example, within the framework of 5G technology, the possibility of using non-orthogonal multiple access (NOMA) is being explored [1,2], which implies ranging and differentiating subscriber signals by power. However, from the point of view of the physical properties of electromagnetic waves, in recent years, the orbital angular momentum (OAM) of EM waves, which defines the vortex dislocation of the signal wavefront, has been of great interest to researchers. This physical parameter was determined 30 years ago and has since found many applications, including also telecommunication applications in both optical and radio communication systems.

As it is known from classical and quantum physics, EM waves (and photons) carry both energy and momentum. The total momentum consists of momentum *P* and angular momentum *L*. In particular, angular momentum has an additional component related to polarization, spin angular momentum (SAM), and the other component related to a spatial field distribution is called orbital angular momentum (OAM). In an optical vortex, the planes of the constant phase of electric and magnetic vector fields form twists or spirals that move in the direction of propagation. That is, the wavefront of the signal obtains a continuous helical structure rather than a set of parallel planes. Wave vorticity is not related to polarization and is another physical property of EM waves. Vortex is characterized by a number called a topological charge (or order) that represents the amount of turns per wavelength of the signal. The application fields of OAM in communication are vast, although there are some issues that need to be solved before full deployment of OAM-based systems. There are some unresolved problems in OAM generation and reception, as well as in practical implementation of related technologies, e.g., mode-hopping spread spectrum (MHSS), which is now under consideration in several research projects [3]. Taking full advantage of the OAM photon dimension to modulate or multiplex data can significantly improve the information capacity of a single photon, which can increase the bandwidth of single wavelength and single mode fiber.

One of the major challenges in the development of OAM-based communication systems is the generation of waves with helical wavefronts. The two main approaches for OAM optical signal generation are free-space optics and fiber-optic technology. Regarding free-space solutions, we can distinguish the following approaches: cylindrical lenses, spiral phase plates (SPP), holographic gratings, spatial light modulators (SLM), metamaterials, liquid crystal q-plates, computer-generated holograms etc. If you want to use these methods to generate OAM beams, you need to use supplementary tools; this is related to the spatial conversion of light and the coupling of optical beams from free space to optical fibers, which leads to problems arising from the complicated procedure of stabilization and alignment of the applied optomechanical devices, as well as their sensitivity to vibration, high cost, and high precision equipment. It is also worth noting that these tools can only be used in laboratory conditions. These methods are described in detail for example in [4,5]. Thus, the task of generating OAM beams in free space optics is generally solved, but it is not clear how to apply free space solutions to already deployed fiber links or future fiber systems.

In addition to free-space optics, several methods have been developed to generate OAM beams directly in the optical fiber. Four main approaches of the research in this area include (1) chiral fibers (including chiral fibers with Bragg gratings), (2) microstructured fibers, (3) photonic lanterns and (4) long-period fiber gratings (LPFGs). We will discuss these methods in more detail.

Helical Bragg grating is one of the simplest and obvious technical ways to create an optical fiber OAM beam. In [6] an optical vortex (OV) generation method is proposed using a helical fiber Bragg grating (H-FBG), which is written directly in a few-mode fiber (FMF). Spiral modulation of the refractive index is achieved by rotating the fiber under one-sided ultra violet radiation using a single-phase mask (in theory). The reflective properties of H-FBG are analyzed analytically. It has been shown that stable OVs can be achieved with precise fiber construction and that the OV order can be tuned by adjusting the resonant wavelength and controllability of H-FBG.

Bragg gratings consisting of a multimode fiber can excite a higher-order linear polarized (LP) mode (*l* > 1). Due to the helical structure of these grating arrays, higher-order modes can be generated with a simple laser model described in [6]. It is also shown in [7] that when using multimode fiber, the conversion of optical fibers with different topological charges: 0 → ±1, ±1 → 0, 0 → ±2, 0 → ±3 with efficiency up to 97% can be achieved. According to [7], one can simply increase the number of fiber modes by changing the fiber parameters in order to excite a higher-order topological charge. For example, an increase in the diameter shown in [7] can enlarge the fiber core size providing the support of the higher-order modes. However, it is not theoretically clear from [6] and [7] how this grating provides proper mode coupling between fundamental and high-order modes.

OAM modes generation methods using conventional quartz fibers typically use additional devices such as polarization controllers (PCs) and mode splitters [8]. However, achieving a specific OAM mode by configuring PCs in all-fiber optic systems is often too difficult. It is even more convenient if you can use a dedicated fiber-optic device to couple the main (fundamental) mode to the desired OAM mode directly. Several studies have shown that the possibility of exciting OAM modes directly with the help of a special microstructured optical fiber construction that is under investigation.

The fiber design utilizes two basic operating principles to convert the fundamental mode to higher-order OAM modes. One of them is developed on the basis of the coupled modes theory [9]. The authors proposed and investigated a tunable fiber optic microstructure to create different OAM modes through modeling based on the coupled modes theory. Microstructured optical fiber consists of a high refractive index ring and a hollow core surrounded by four small air holes as shown in [9]. The hollow core and surrounding four air holes are infiltrated by optical functional material whose refractive index can be physically changed (e.g., by voltage), causing conversion between the polarized fundamental mode and different OAM modes in different cycles of a high refractive index ring with normal operating wavelength.

Another way to generate the OAM mode in-fiber is to mimic the element of the repetitive spiral phase [10,11]. This method typically uses multicore fiber (MCF) [10] or photonic crystal fiber (PCF) [11]. For MCF, phase transformation is a function of the core refractive index. The phase difference between the nearest cores is exactly 2π*l*/*N*, where *l* is the order of the required OAM order and *N* is the number of MCF cores. With such a phase differentiation, when spatial phase modulated beams are repeatedly connected to a ring core fiber (RCF), the OAM with the order of *l* is effectively generated.

The third approach is based on the use of so-called photonic lanterns (PLs)—spatial mode converters that connect unidirectional single-mode signals from multiple individual waveguide cores into a single multimode waveguide, fabricated either by using optical fibers or planar waveguides [12]. PL can be classified as a spatial multiplexer for spatial multiplexing systems because PL can be considered as an *N*-mode multiplexer, where the *N* is the SMF number used to manufacture the PL, which can actually provide the communication system capacity multiplication by factor *N*. For example, in [13] the authors show a method of creating OAM modes, based on a PL with a center of all threaded rings. The device consists of a 5-mode selective photonic lantern (MSPL), with an effective refractive index profile that is arranged to a ring shape. It has been shown that high-quality OAM beams are generated up to the second-order by simultaneous excitation of the degenerate linear polarized mode pairs of the MSPL.

Regarding fibers maintaining vortex propagation, several approaches have been developed. The earliest was based on a fiber proposed by Ramachandran [14] having a refractive index resembling a coaxial structure. The next approach of the OAM fiber-generation methods was based on the inverse parabolic profile of refractive index [15]. The reverse parabolic reverse grading index (IPGIF) fiber has been proposed for robust transmission of cylindrical vector modes as well as integer OAM carrying modes. Large isolation of an efficient index between vector modes in the mode group LP_11_ {TE_01_, HE_21_, TM_01_} (>2.1 × 10^−4^) is numerically and experimentally confirmed in this fiber in the whole C-band as well as the possibility of OAM transmission of +/−1 orders for distances up to 1.1 km. The authors also provide simple design optimization rules for redefining fiber parameters.

Finally, the last method considered was based on the LPFGs [16]. It has been shown that the OAM signal can be generated via twisting a strong modulated LPFG written in a four-mode fiber (4MF). With a special design and optimization of the procedures of CO_2_-laser irradiation, an LPFG with strong period deformation is achieved in the 4MF. Based on this LPFG, one can directly convert the fiber fundamental mode (LP_01_) to the high-order LP core mode (LP_21_) with an efficiency of 99.7% and then transform the LP_21_ mode into a high-order vortex mode (±2 order).

The main disadvantage of the considered methods of generating OV is their relative complexity, which implies more precise doping and, for example, additional equipment for rotating the fiber when applying Bragg gratings or manufacturing PLs, as well as low modal purity due to the need to use precise equipment. For example, fiber methods such as LPFGs require real-time fiber rotation and other fiber optic tools such as PCs.

In our paper, we will focus on the fiber-optic method for OAM modes generation, based on the application of a chiral (vortex) fiber Bragg grating (ChFBG). The proposed method allows the creation of complete passive fiber-optic devices that can be used in telecommunication systems as OAM mode generators aiming to increase the throughput of communication channels by OAM modes multiplexing. For example, such modes can be applied for radio-optical systems implementation using both optical and radio vortexes [17,18,19] or in quantum computing [20].

## 2. The Theoretical Model for a Chiral (Vortex) Fiber Bragg Grating (ChFBG)

As mentioned above, the OAM signal generation in an optical fiber is currently an underinvestigated and urgent scientific and technical problem. One of the main goals in this field of research is to develop a full fiber-optic passive device that does not require any additional optical components such as polarization controllers (PC) in the case of LPFGs [16], mirrors, lenses, etc. In other words, the proposed device should be a complete fiber optic passive element. In this paper, we propose a method for generating a first-order OAM mode based on ChFBG, which in the general case is a continuous diffractive fiber structure with a spiral (helical) shape. Conventional fiber Bragg gratings, represented by a discrete set of fingers [21], can be defined, for example, using an approach similar to the tunneling effect description in quantum mechanics [22]. However, the most common approach to describe FBGs is the coupled modes theory. The OAM mode generation in this context means the conversion of the fundamental mode into a higher-order mode: in terms of vortex mode generation, it is necessary to achieve the highest possible conversion of the OAM_0_ mode into OAM_1_; therefore, the theoretical model of the ChFBG is based on the aforementioned coupled modes theory, considering ChFBG as a type of a mode-coupling device. It is known that the existence of vortex modes requires a few-mode fiber-optic regime since any non-zero order OAM mode is represented by a superposition of the degenerated TE (transverse electric) and TM (transverse magnetic) modes [23]. Thus, since G.652 fiber is multimode in the first spectral window only, the operation in the optical C-band (1530–1565 nm) requires special few-mode fibers, e.g., the step-index FMF by the Optical Fiber Solutions (OFS) company. For the fiber considered, the approximation of a weakly guiding fiber is applicable, i.e., a fiber in which the difference between the refractive indices of the core and the cladding is less than 1% (note that the most commercially available fibers are considered as weakly guiding). In this case, the apparatus of Bessel functions and LP-modes, mentioned in previous section, can be used to describe the mode composition (including OAM modes) of the optical field propagating through the optical fiber [23]. Hence, using the weakly guiding fiber approximation and Bessel-function formalism, the full-fiber OAM-generation problem statement can be formulated as follows: it is necessary to develop a grating that transforms the zero-order OAM mode (with flat wavefront—LP_01_):

$$E_{01}(r,\varphi ,z)=e^{-i\beta_{01}z}\frac{J_{01}\left(u_{01}\frac{r}{a}\right)}{J_{01}(u_{01})},0\leq r\leq r_{co}$$

into the first-order OAM mode (superposition of two spatially shifted LP-modes: ${\mathrm{LP}}_{11}^{e}+i{\mathrm{LP}}_{11}^{o}$):

$$E_{11}(r,\varphi ,z)=e^{i\beta_{11}z}\frac{J_{11}\left(u_{11}\frac{r}{a}\right)}{J_{11}(u_{11})}e^{i\varphi },0\leq r\leq r_{co}$$

where *r*_co_ is the core radius (since the grating only exists in the fiber core), *J*_01_ and *J*_11_ are Bessel functions of 01 and 11 orders, respectively. The parameters *u*_01_ and *u*_11_ will be described further in the text.

**Figure 1:**
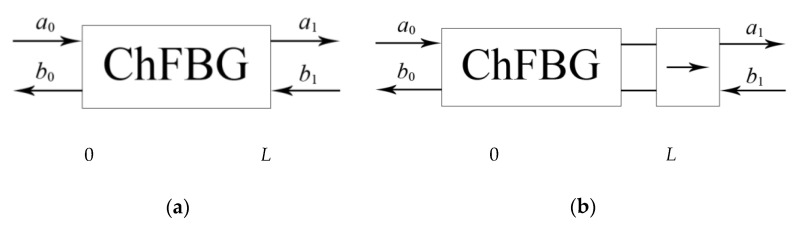
The schematic representation of the chiral (vortex) fiber Bragg grating (ChFBG): (**a**) in the form of a four-pole circuit; (**b**) with isolator for reflection mitigation.

From a functional point of view, the ChFBG can be considered as a four-pole circuit with a length from 0 to *L* (Figure 1a), described by a transfer matrix *T*; where *a*_0_ is the incident radiation—Gaussian-like incoming field—LP_01_ (OAM = 0); *b*_0_ is the reflected radiation—the aforementioned superposition ${\mathrm{LP}}_{11}^{e}+i{\mathrm{LP}}_{11}^{o}$ (OAM = 1); and *a*_1_ is radiation passed through the grating; *b*_1_ is the radiation incident on the grating from the receiver side; in the general case, radiation *b*_1_ arises due to inevitable Rayleigh scattering or in case of duplex communication. Such radiation provides the generation of a reflected signal in the *b*_1_ direction, which is an undesirable circumstance, since additional low-power radiation will appear in the grating, i.e., performing additional noise and re-reflections. One can easily remove undesirable *b*_1_ by placing an optical isolator behind the grating (Figure 1b).

**Figure 2:**
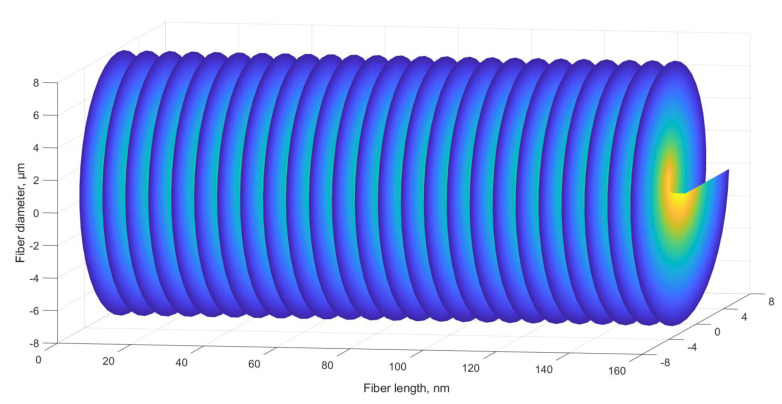
The schematic shape of the regular ChFBG.

The shape of the ChFBG is presented in Figure 2.

Thus, expressing the radiation at the end of the grating (*z* = *L*) through the radiation at the beginning of the grating (*z* = 0), it can be defined as column vectors:

(1) $$\left(\begin{array}{l} a_{1}\\ b_{1} \end{array}\right)=T\left(\begin{array}{l} a_{0}\\ b_{0} \end{array}\right)$$

The transmission matrix *T* is represented as follows:

$$T=\left(\begin{array}{cc} T_{11} & T_{12}\\ T_{21} & T_{22} \end{array}\right)=\left(\begin{array}{cc} w & z\\ z* & w* \end{array}\right)$$

In order to find the elements of the *T* matrix, we use the well-known approach described in [24,25], namely, the coupled modes theory [26,27], together with the model of field coupling under arbitrary perturbations of the refractive index of the fiber, considered in [28]. According to these approaches, field reflection and transmission coefficients are complex numbers determined through the matrix *F*, used in [27] to describe radiation of arbitrary diffraction structures, and associating radiation at the beginning of the grating *E*(0) with radiation at the end of the grating *E*(*L*): *E*(0) = *F·E*(*L*). In contrast to the approach proposed in [27], matrix *T* is defined by connecting the reflected and transmitted radiation through the incident radiation. Therefore, the matrix *T* is the inverse of the matrix *F*:*T* = *F*^−1^. According to the matrix-converting rule and taking into account that determinant of the *F* matrix is unitary, we obtain:

(2) $$T=\left(\begin{array}{cc} T_{11} & T_{12}\\ T_{21} & T_{22} \end{array}\right)=\left(\begin{array}{cc} F_{22} & -F_{12}\\ -F_{21} & F_{11} \end{array}\right)$$

Therefore, taking into account (1) and (2), we can state the relation between refracted and incoming light in the general case:

(3) $$\begin{array}{l} a_{1}=T_{11}a_{0}+T_{12}b_{0}=F_{22}a_{0}-F_{12}b_{0},\\ b_{1}=T_{21}a_{0}+T_{22}b_{0}=-F_{21}a_{0}+F_{11}b_{0} \end{array}$$

Assuming that *b*_1_ = 0 (no backward radiation), it can be found from (3) that the equations for coupling modes in the case of a ChFBG remain the same as for a regular FBG, differing in the coupling coefficient only, therefore, with respect to [27] we can obtain:

(4) $$\begin{array}{l} b_{0}=wa_{0}=a_{0}\frac{F_{22}}{F_{11}}=a_{0}\frac{-k_{ab}\sinh(\gamma L)}{\gamma \cosh(\gamma L)+i\Delta \beta \sinh(\gamma L)},\\ a_{1}=z*a_{0}=\frac{1}{F_{11}}a_{0}=a_{0}\frac{\gamma e^{-i\beta_{0}L}}{\gamma \cosh(\gamma L)+i\Delta \beta \sinh(\gamma L)} \end{array}$$

where the phase coefficient difference Δβ for counter-propagating modes is equal to [25]:

$$\Delta \beta =\beta_{01}+\beta_{11}-2\beta_{0}$$

In relation (4), the complex coefficient *w* refers to the reflection coefficient, z* is the transmission coefficient, β_0_ is defined below and depending on Λ—the grating period, defined as Λ = λ_B_/2*n*_0_, where λ_B_ is the Bragg reflection wavelength; and β_01_ and β_11_ are the phase coefficients of the incident (OAM = 0) and reflected modes (OAM = 1), respectively. The parameter γ is defined as $\gamma^{2}=k_{ab}^{2}-\Delta \beta^{2}$, while *k_ab_* is the complex overlap integral (which specifies the coupling coefficient between modes) determined by the inhomogeneity of the refractive index δn [28]:

(5) $$k_{ab}=\frac{i{\omega \varepsilon }_{0}}{2}\int_{-\infty }^{\infty }\int_{-\infty }^{\infty }\delta n^{2}(r,\varphi )\vec{E_{a}^{*}}(r,\varphi )\cdot \vec{E_{b}}(r,\varphi )drd\varphi$$

or, in the case of LP modes:

(6) $$k_{ab}=\frac{i{\omega \varepsilon }_{0}}{2}\int_{-\infty }^{\infty }\int_{-\infty }^{\infty }\delta n^{2}(r,\varphi )\vec{E_{11}^{*}}(r,\varphi )\cdot \vec{E_{01}}(r,\varphi )drd\varphi$$

It is easy to show that ${\left|w\right|}^{2}+{\left|z\right|}^{2}=1,$ i.e., the sum of the squares of the absolute values of the reflection and transmission coefficients, is equal to 1. Note that, in the absence of field coupling caused by the transverse refractive index perturbation, the ChFBG acts as a regular FBG without changing the structure of the reflected field.

**Figure 3:**
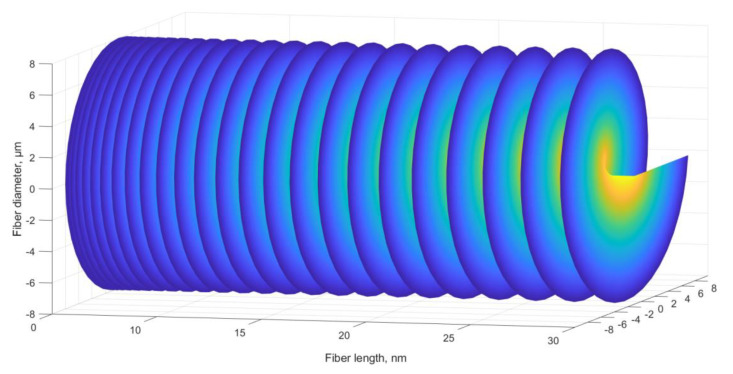
Chirped ChFBG with a linear increase in the lattice period.

Finally, the refractive index of the ChFBG is defined as follows:

(7) $$n(r,\varphi ,z)=n_{0}+\Delta n\left[e^{-i\beta_{0}z}\cdot g(z)\cdot f(r)\cdot e^{im\varphi }+c.c.\right]$$

where Δn is the modulation amplitude of the refractive index (which must be lower than in regular FBG, see below), *m* is vortex order, β_0_ = 2π/*y*(*z*), *y*(*z*) is the chirp function (Figure 3), used to make the ChFBG broadband and which in the general case has an arbitrary form.

Note that the azimuthal position Δφ of the grating finger is defined by Δφ=(2π/Λ)⋅Δz. Thus, for linear chirp we can write *y*(*z*) = Λ + Λ_0_*z* (without chirp—*y*(*z*) = Λ); *g*(*z*) is the apodization function, used to narrow down the reflection spectrum and change its shape; in our case *g*(*z*) is equal to 1 (there is no apodization), *f*(*r*) is the radial function, determining the radial refractive index perturbation (because the grating is uniform in the azimuth), since the degree of mode coupling depends on *k_ab_* according to (5).

**Figure 4:**
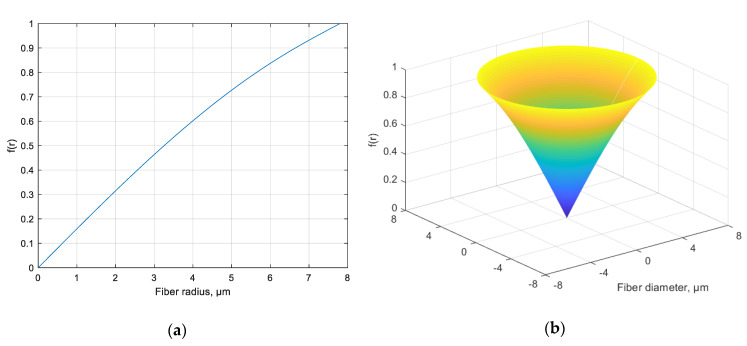
(**a**) The shape of the radial function *f*(*r*) according to (8), describing the transverse profile of the refractive index of the grating finger; (**b**) 3D image of a homogeneous finger with a given profile (in case of non-chiral regular grating, normalized units).

**Figure 5:**
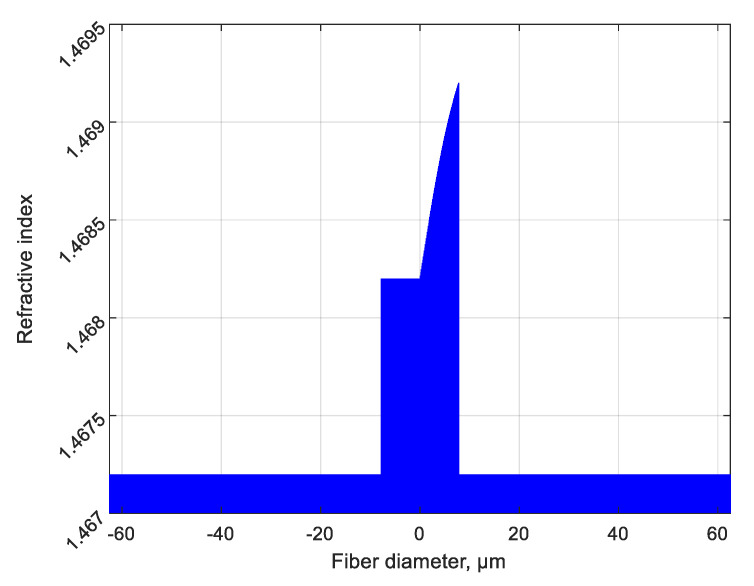
The refractive index profile of the ChFBG’s cross-section.

According to the coupled modes theory, mode coupling occurs at a non-zero functional transverse perturbation of the refractive index (according to [28]). Since in a classical (non-vortex) FBG, there is no functional transverse perturbation (as a function of the fiber radius or azimuth angle), but only a quantitative increment of the refractive index Δn exists, the overlap integral (5) is not zero only in the case when *a* = *b*, i.e., when the incident and the reflected modes are the same, and equals to zero otherwise. Hence, this follows the conclusion that was theoretically substantiated in [25]: the conventional FBG does not lead to mode coupling. However, it is obvious that in order to convert the OAM_0_ mode to the OAM_1_ mode, the overlap integral between these modes must be non-zero, and in the case of orthonormal signals, it should be close to 1 (which means 100% energy transfer from the OAM_0_ mode to the OAM_1_ mode). In other words, one needs to make orthogonal modes non-orthogonal to increase power coupling between them. Based on these considerations, it can be shown that the radial function *f*(*r*) can be defined as follows:

(8) $$f(r)=\sigma \frac{J_{11}\left(u_{11}\frac{r}{r_{co}}\right)}{J_{01}\left(u_{01}\frac{r}{r_{co}}\right)}$$

where σ is the normalizing coefficient, and the factors *u*_01_ and *u*_11_ are the roots of the characteristic equation for a particular type of fiber in which the grating is written; these factors can be calculated easily for any step-index fiber. Figure 4 shows the shape of the function (8), which describes one period of the considered grating; the corresponding refractive index profile of one finger of the ChFBG is shown in Figure 5. This function, according to (6), theoretically provides an absolute magnitude of the overlap integral equal to 1.

Considering that the magnitude of Δn should be around 10^−3^ (which will be explained below), it can be defined as:

(9) $$n^{2}∼n_{0}^{2}+2n_{0}\Delta n\cdot e^{-i\beta_{0}z}\cdot f(r)\cdot e^{im\varphi }$$

where $\delta n^{2}(r,\varphi )=2n_{0}\Delta n\cdot f(r)\cdot e^{im\varphi }$. Then expression (6) can be overwritten as follows:

(10) $$k_{ab}=i\omega \varepsilon_{0}n_{0}\Delta n\cdot \int_{0}^{2\pi }\int_{0}^{r_{co}}e^{im\varphi }f(r)\cdot \vec{E_{11}^{*}}(r,\varphi )\cdot \vec{E_{01}}(r,\varphi )drd\varphi .$$

The factor $e^{im\varphi }$ describing the chirality of the grating provides the integral (10) to be non-zero which means the non-zero probability of conversion of LP_01_ mode to OAM_1_ mode. In this case, *m* should be equal to 1. Note that the reflection coefficient *w*, according to (4), upon apodization has a dependence on *z*, i.e., *w* = *w*(*z*). The amplitude of the reflected mode in the case of grating apodization can be defined as [28]:

(11) $$b_{0}\approx e^{i\beta_{11}L}\int_{0}^{L}w(z)e^{-i\left(\beta_{01}-\beta_{11}\right)z}dz$$

where the reflection coefficient *w*(*z*) is determined by (4)–(9). Otherwise, the amplitude of the reflected filed has a longitudinal phase shift only.

**Figure 6:**
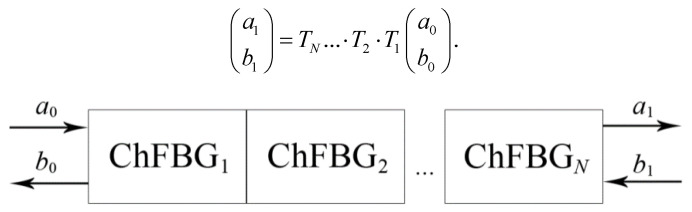
Matrix model of irregular ChFBG.

The matrix approach considered in this work is convenient for the analysis of irregular and/or cascade gratings, that can be used, e.g., for broadband operation; in this case, it is possible to split a complex grating into *N* regular sections (Figure 6), each of which is described by its own matrix *T_i_*, and the resulting field can be expressed as a product of the matrices:

$$\left(\begin{array}{c} a_{1}\\ b_{1} \end{array})=T_{N}\ldots \cdot T_{2}\cdot T_{1}(\begin{array}{c} a_{0}\\ b_{0} \end{array}\right)$$.

The reflectivity of the ChFBG depends on several factors, for example, the magnitude of the refractive index modulation Δn, number of periods *N*, etc. However, mode coupling depends mostly on transverse perturbation of the refractive index. In the following section, ChFBG properties are investigated by numerical simulation.

## 3. Numerical ChFBG Analysis and Fiber Design

**Figure 7:**
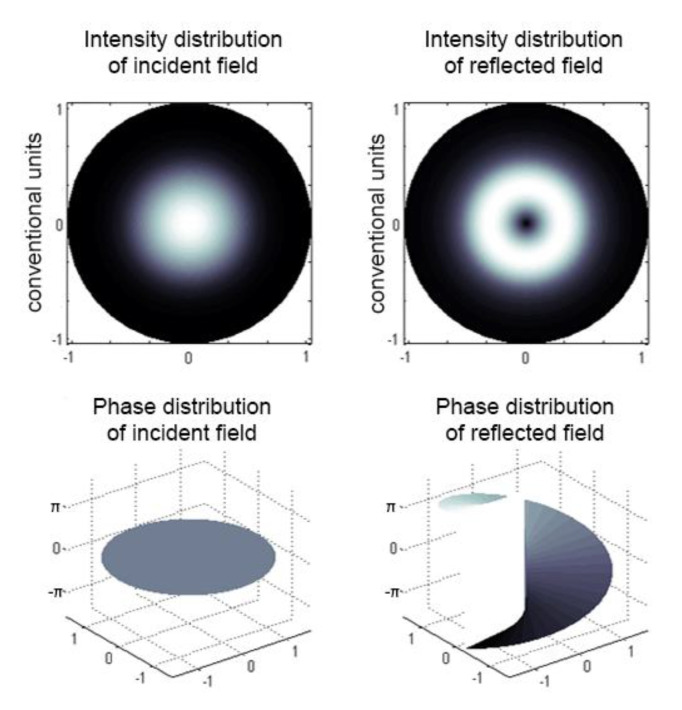
Reflected and incident grating field.

The proposed ChFBG is designed to transform an incident field with a plane wavefront into a field that carries the OAM. Figure 7 shows the incident and reflected fields of the considered grating.

Analyzing Figure 7 we can assert that the vortex structure of the field is formed in the reflected radiation. It can be shown that counter-propagating modes—incident plane wave and reflected OAM wave—are still orthogonal and can be separated. One can separate the reflected field from the incident one, e.g., using a mode splitter [29].

**Figure 8:**
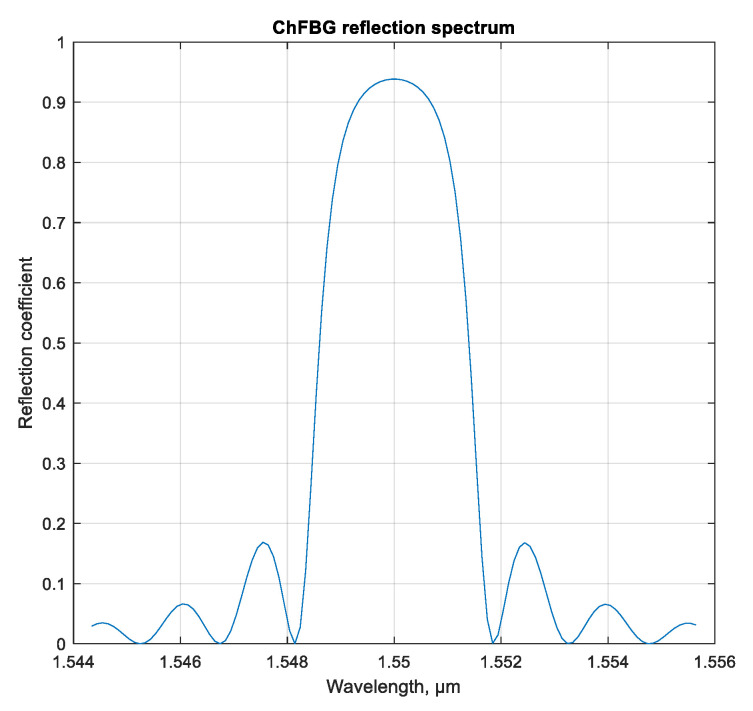
Spectral dependence of the reflection coefficient of the ChFBG.

According to expression (4), it is possible to construct the reflection spectrum of the ChFBG, shown in Figure 8.

As is clear from Figure 8, the considered grating has a reflection coefficient around *R* = 0.95 at 1550 nm, however, also significant side lobes occur. If necessary, the spectral characteristics of the ChFBG can be changed by chirping or using the apodization function, as mentioned above. Note that in this calculation we used Δ*n* = 0.003 and 1000 grating periods.

**Figure 9:**
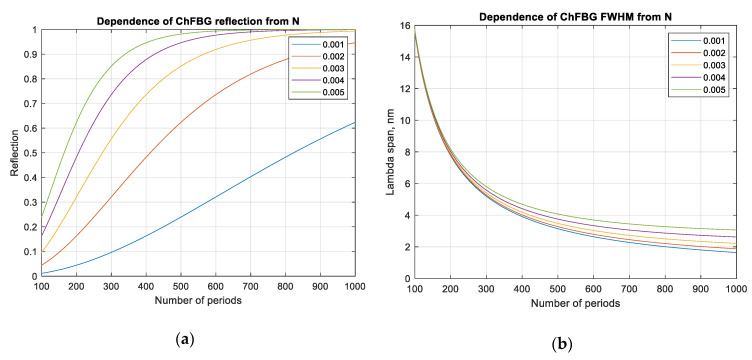
Relation of the reflection coefficient and spectrum of the ChFBG: (**a**) refractive index versus the number of periods (fingers), *N*; (**b**) width of the reflection spectrum vs. *N* of the grating at different values of the amplitude of the refractive index induced modulation.

Since in order to apply the mathematical apparatus of Bessel functions to define OAM modes, it is necessary to remain within the weakly guiding fiber approximation (Δ*n* < 1%), the amplitude of the induced grating modulation should be relatively low compared to classical FBGs (about 0.005 for the ChFBG versus 0.01 for classical FBG). In this regard, the reflection coefficient, according to (4), can be improved by increasing the number of grating periods *N* (Figure 9).

According to Figure 9, the grating parameters can be selected according to the initial requirements or purposes, for example, one can easily find Δ*n* and *N* values for a given reflection coefficient, or choose Δ*n* and *R* for a specific *N*, etc. It should be noted that the overlap integral (10) has only a phase incursion $e^{-i\beta_{0}z}$ along the *z* axis, and the integral’s absolute value depends only on the transverse coordinates. This means that the efficiency of the first-order OAM mode generation from the fundamental mode does not depend on the number of grating fingers, but depends on the transverse index perturbation. This statement is also confirmed, for example, by the fact that only one spiral phase plate is sufficient for the vortex radio beam generation [30], and for the free-space optical vortex beam—a single diffractive optical element is used to generate the OAM signal. In this regard, the number of grating periods determines not the efficiency of the OAM mode generation, but the efficiency of its reflection.

One of the key practical issues is the method of manufacturing the grating under consideration. As is known, there are many ways to create fiber Bragg gratings: using phase masks [31] and Talbot interferometers [32], using a Lloyd prism [33], etc. The most promising technology for writing ChFBG seems to be a step-by-step method with simultaneous fiber rotation, but this approach requires a high precision of installation and control of both the grating pitch and the degree of its twist. The required low amplitude of the induced modulation of the refractive index makes the task easier in terms of the laser intensity and the required fiber photosensitivity, but it requires a relatively large number of grating periods. Moreover, the question of the temperature dependence of such a grating and changes in the structure of the reflected field under its influence remains unexplored.

It is obvious that after the considered mode generator, the optical signal must be coupled to the corresponding guiding medium. It was mentioned above that a standard stepped optical fiber is not multimode in the operation wavelength range. Moreover, in the Introduction some works devoted to the development of fibers that support the propagation of vortex optical radiation were considered. It was previously mentioned that the generated OAM optical signal can be branched off using an LP-mode splitter. The authors of this work have previously proposed an optical fiber that supports the propagation of optical vortexes [34]. The proposed fiber design is based on microstructured fibers. It is assumed that the induced chirality on the optical fiber with a special design—microstructured core—will allow us to form the desired order vortex (OAM mode), while the chirality on the standard fiber—the core surrounded by one outer continuous shell—can be used as a “translator”, i.e., vortex-maintaining fiber for relatively long distances (note that ChFBG means not twisted fiber with a chiral diffraction structure in it; meanwhile, in [34] we consider the whole twisted fiber). For this purpose, a modification of the rotor of the exhaust tower was carried out: engine rotation speed before modernization was 3 rpm. After refinement, it can now be accelerated to 66 rpm with a drawing speed of 1 m/min. As a result, 2 regimes of optical fiber twisting during the drawing from the workpiece are provided: a step with 10 rpm and a step with 66 rpm. In any case, the engine rotates the workpiece slowly to compensate environmental side effects, so this controllable rotating modification should provide chiral fiber with precise twisting step. Thus, the following scenarios for the implementation of specialized optical fibers for the generation and transmission of OAM are considered.

The first one is an FMF with a step-index refractive index profile and strong induced chirality. We start with the profile of a conventional single-mode fiber (Rec. ITU-T G.652). Next, a theoretical calculation and simulation of fiber properties are carried out in COMSOL Multiphysics in such a way that, with a typical core diameter of 8.3 μm and a correspondingly increased Δ*n*—the difference between core and cladding refractive index (thus increased numeric aperture, *NA*), the desired modes LP_01_ and LP_11_ satisfy the cut-off condition. Practically, the cut-off wavelength can be shifted by doping GeO_2_ core of the fiber. An alternative approach is to slightly increase both the diameter of the fiber core and Δn. In turn, strongly induced chirality will allow these three modes to be “folded” into the desired 1st order OAM. The induced chirality by the aforementioned drawing process modification adds to the desired mode coupling.

**Figure 10:**
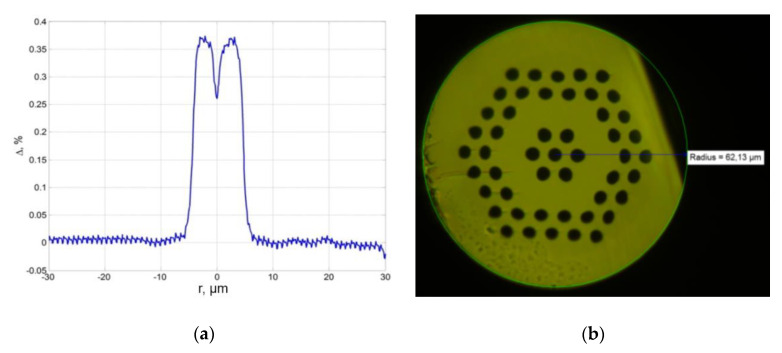
(**a**) The relative Δ*n* between core and cladding for the proposed fiber. One can obtain a ring-structured profile of refractive index; (**b**) the proposed micro-structured fiber with ring-shape hexagonal geometry [34].

The second approach is based on microstructured optical fibers with geometry that mimics the structure of a ring-cored fiber and also has an induced chirality (Figure 10a,b). It is known that fiber with 5.6 μm (“thickness” 1.8 μm) ring core is an applicable solution for the OAM generation. The proposed micro-structured optical fiber is a replica of this design, where the ring is reproduced by the corresponding positioning of the fiber capillaries. In turn, strongly induced chirality should only enhance the effect of the generation and/or maintenance of an OV.

Thus, the considered method of optical OAM modes generation should be used in combination with guiding structures that support the propagation of vortex signals. This full-fiber optic segment can be used in future OAM-based mode-division multiplexing (MDM) systems [35] and radio-over-fiber [36] networks as well.

## 4. Sensor Application

**Figure 11:**
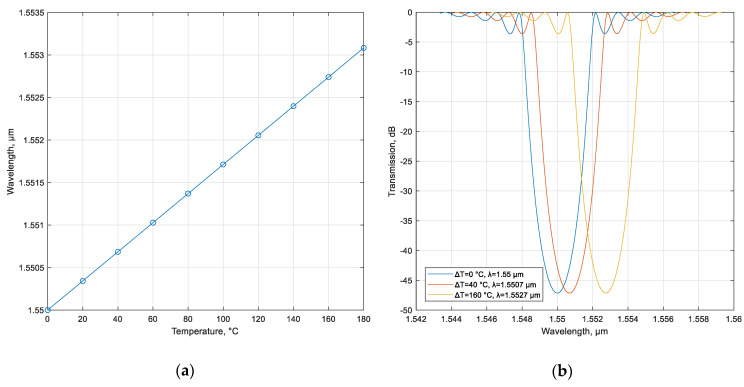
(**a**) ChFBG wavelength as a function of temperature change; (**b**) ChFBG transmission as a function of temperature change for three temperature increments: 0 °C, 40 °C, and 160 °C.

It is well known that FBGs can be used as sensors of many physical parameters. It can be shown that the proposed grating is also sensitive to temperature and strain, which manifests itself in a corresponding change in the reflection wavelength:

(12) $$\frac{\Delta \lambda_{B}}{\lambda_{B}}=(1-p_{e})\varepsilon +(\alpha_{\Lambda }+\alpha_{n})\Delta T$$

where *p*_e_ is the strain optic coefficient, α_Λ_ is the thermal expansion coefficient (which is 0.55 × 10^−6^/°C for silica), α_n_ is the thermo-optic coefficient (1.05 × 10^−5^/°C for silica) [37], ε = Δ*L*/*L* is strain, *L* is ChFBG length, Δ*L* and Δ*T* are length and temperature increments, respectively. It should be noted that, in this case, the chirality of the grating does not depend on the wavelength in terms of the topological charge—OAM order (i.e., when the wavelength changes due to heating or stretching of the ChFBG, its chiral nature will be conserved; therefore, the OAM order does not depend on external factors). Since the distance between any two adjacent point of grating fingers with the same spatial phase Δφ remains unchanged and equal to Λ, environment changes will lead to proportional changes in grating period along the whole ChFBG. The proposed ChFBG, as well as chiral fiber gratings [38], can be used as sensors for temperature, pressure, etc. In [38] it is proven experimentally that chiral fiber gratings can act like sensors. However, in contrast to reference [38], in which authors use high-temperature silica fiber, the proposed ChFBG is supposed to be written in a conventional silica fiber with the temperature parameters listed under expression (12). Thus, we obtain the following dependences for the reflection wavelength on temperature (Figure 11). The results obtained are in good agreement with classical FBG sensors [24]. In contrast to classical FBGs, the proposed grating is not only an effective sensor of physical parameters, but also has an important advantage—invariance of the OAM order (chirality) of the reflected mode to changes in the physical parameters, since as was mentioned above grating chirality remains constant with changes in grating period caused by temperature or/and strain. Thus the main advantage of the proposed solution over the device presented in [38] or in [24] is that it can also replace (or supplement) the complex arrays of fiber Bragg gratings in so-called addressed sensor systems.

**Figure 12:**
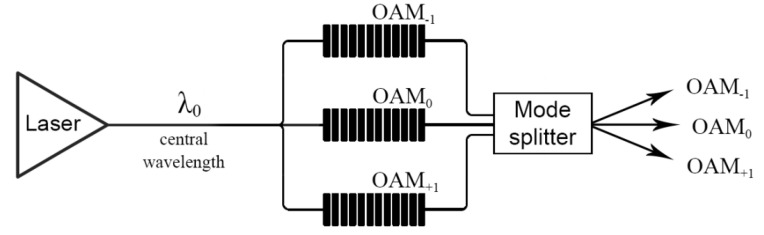
Schematic example of an addressed sensor system based on an array of single-frequency ChFBGs: A coherent optical signal from a laser at a wavelength λ_0_, which is a central wavelength for all gratings, enters an array of three ChFBGs, which form signals with orbital angular momentums OAM_−1_, OAM_+1_ and OAM_0_. The signals from the sensors can be separated using a mode splitter and directed to further processing (the photodetector is not shown in the figure).

One of the main problems of such systems is addressing, i.e., discrimination between signals received from different gratings operating at the same central wavelength or in the case when a change in the measured physical parameters leads to overlapping of the reflection spectra of different gratings. To solve this problem, the authors of [21] proposed a method for addressed sensors based on the use of two-frequency gratings, the difference between the main reflection frequencies of which remains constant for any change in the measured physical field and is thus a unique sensor address in complex systems. The ChFBG, proposed in this article, can be used as both an alternative and an addition to this system: using a ChFBG that generates OAM signals of +1 and −1 orders (the zero order of OAM is generated by conventional FBG), it is possible to construct sensor systems using only one wavelength, but provide addressing due to the spatial distribution of the optical field. When polling such sensors, the signals will (or can) have the same frequency, but be spatially orthogonal and, therefore, such signals can be separated using, for example, mode splitters or other passive fiber-optic components (Figure 12).

Thus, the use of gratings for three OAM orders (−1, 0, +1) will increase the capacity of sensor systems threefold, but, however, requires the use of additional passive optical components for mode separation. The manufacture of apodized or chirped ChFBGs is a complex technical problem, but it will make it possible to develop sensor systems based on narrow-band (or broadband) gratings that change the spatial distribution of the reflected field. Thus, the use of ChFBG in sensor systems allows us to develop addressed sensor arrays with an address in both the frequency and spatial domains, which makes it possible to increase the number of sensors without using additional active equipment.

## 5. Conclusions

In this paper, we present a theoretical model of a chiral fiber Bragg grating based on the coupled modes theory modes and scattering matrices. The proposed model allows us to study the characteristics of a grating with arbitrary apodization and chirping functions, obtaining a ChFBG with the required spectral characteristics; additionally, it can define complex (cascade) gratings. The radial perturbation function of the refractive index provides up to 100% of the theoretical efficiency in the OAM_1_ to OAM_0_ mode conversion. Moreover, based on the analysis of the disadvantages of existing methods for optical vortex signal generation, a new optical fiber is proposed, which is a chiral microstructured optical fiber that mimics a ring-core structure and allows the formation of first-order OAM modes. Since the cross-section of the fiber is a periodic structure (formed by capillaries), it can be considered as a photonic crystal and, therefore, as a spatial-phase filter for the generation of vortices with the desired order. As a vortex-maintaining fiber, we propose a strongly chiral multimode fiber of size 100/125 μm with low modal dispersion that allows the vortex structure of the optical field propagating over relatively large distances to be maintained. These novel fibers can be applied in future communication systems, e.g., in the radio-over-fiber backbone infrastructure. Thus, in this paper, a full-fiber vortex generation method and vortex maintaining fiber have been proposed. New application of the results obtained in this work as address sensors with an address in the form of a constant topological charge in multi-sensor systems for monitoring various physical fields is also proposed.
